# Antimicrobial Carvacrol Incorporated in Flaxseed Gum-Sodium Alginate Active Films to Improve the Quality Attributes of Chinese Sea bass (Lateolabrax maculatus) during Cold Storage

**DOI:** 10.3390/molecules24183292

**Published:** 2019-09-10

**Authors:** Shiyuan Fang, Qianqian Zhou, Yan Hu, Feng Liu, Jun Mei, Jing Xie

**Affiliations:** 1 College of Food Science and Technology, Shanghai Ocean University, Shanghai 201306, China; 2 National Experimental Teaching Demonstration Center for Food Science and Engineering Shanghai Ocean University, Shanghai 201306, China; 3 Shanghai Engineering Research Center of Aquatic Product Processing and Preservation, Shanghai 201306, China; 4 Shanghai Professional Technology Service Platform on Cold Chain Equipment Performance and Energy Saving Evaluation, Shanghai 201306, China

**Keywords:** carvacrol, antimicrobial activity, Chinese sea bass, active coating, shelf life

## Abstract

The objective of this research was to explore the antimicrobial activity and mechanism of carvacrol against *Vibrio Parahemolyticus*, *Shewanella putrefaciens*, *Staphylococcus aureus* and *Pseudomonas fluorescens* and evaluate the effect of the addition of carvacrol/β-cyclodextrin emulsions to flaxseed gum (FSG)-sodium alginate (SA) edible films on the preservation of Chinese sea bass (*Lateolabrax maculatus*) fillets during refrigerated storage. The minimum inhibitory concentration (MIC) of carvacrol against *V. parahemolyticus*, *S. putrefaciens*, *S. aureus* and *P. fluorescens* were 0.5, 0.5, 0.125, and 0.5 mg/mL, respectively. Alkaline phosphatase activity assay, nucleotide and protein leakage, and scanning electron microscope demonstrated that carvacrol damaged the external structure of the tested bacterial cells causing leakage of cytoplasmic components. At the same time, when FSG-SA films containing carvacrol used as coating agents for Chinese sea bass fillets cold storage, FSG-SA films containing 1.0 or 2.0 mg/mL carvacrol could significantly reduce TVB-N content, K-value, the degree of microbial deterioration and maintain quality of sea bass fillets according to organoleptic evaluation results.

## 1. Introduction

Chinese sea bass (*Lateolabrax maculatus*) is recognized as one of the most important mariculture fish in China, and its high protein and low-fat content make it value-added seafood product with increasing demand [1]. However, gutted sea bass is extremely susceptible to endogenous enzymes and exogenous spoilage microflora, leading to lipid oxidation, protein degradation, or decomposition [2]. The short shelf life of refrigerated sea bass is a hinderance to the sales of fresh sea bass, therefore, extending the gutted sea bass shelf life and keeping the freshness would benefit the sea bass farming.

Active coating is an innovative concept that could extend the shelf-life of seafood products and provides microbial safety for consumers [3,4,5]. Materials available for active coating generally include polysaccharides, proteins and lipids. Sodium alginate (SA) is a polysaccharide deprived from brown algae and mainly composed of 1-4 linked α-l-guluronic acid and β-d-mannuronic acid [6]. SA has the advantages of good gel property and film-forming ability, which is suit for preparing SA-based composite films for the preservation of seafood products [7,8,9]. The edible membrane prepared by SA has high CO_2_ transmittance and low O_2_ transmittance, when hydrophilic SA intersects with hydrophobic β-cyclodextrin, its stability is greatly improved and it has long-time treatment [6,10,11]. Contact preservatives applied directly to the food surface are not effective enough to inhibit the food borne pathogenic microorganisms’ growth, which due to the rapid spread of the preservatives into foods and denaturation with the food components resulting in reducing the antimicrobial activity. However, the active packaging containing antimicrobial agents could provide a slow and continuous way for these agents migrating form packaging materials to food surfaces for a long time [12]. Carvacrol is generally recognized as safe (GRAS) defined by the US Food and Drug Administration (FDA), and has been applied to promote the shelf-life and safety of seafoods for its broad spectrum of antimicrobial and anti-biofilm activities [13,14,15]. However, the main problem limiting its application is poor solubility. Many researchers use phospholipid or polysaccharide encapsulated carvacrol to overcome these problems [16,17]. Carvacrol can be incorporated into biopolymer materials, either as an independent film or coated in synthetic packaging materials. It should be noted that the concentration of carvacrol in this system should be high enough for antimicrobial impact where necessary [18]. β-cyclodextrin (βCD) is also recognized as a GRAS food additive and is a cyclic oligosaccharide with truncated cone shapes and hydrophobic cavities that can completely or partially nanoencapsulates geometrically compatible hydrophobic compounds [19,20]. The access of oxygen and light to the reactive sites of the guest compounds is much lowered, therefore, the oxidative stability of the nanoencapsulated labile compounds is enhanced. Furthermore, the high number of the hydroxyl groups of the cyclodextrin structure (comprise of 6–8 glucopyranose units) allows significant enhancement of the apparent water solubility of hydrophobic compounds entrapped into the cyclodextrin cavity. This also determines enhanced bioavailability and controlled release properties [21,22].

To our knowledge, no studies have been conducted to investigate the preservative effects of SA coating enriched with carvacrol/βCD emulsions on the qualities of fish muscle. Thus, the dual aim of this work is (i) to evaluate the antimicrobial activity of carvacrol to some specific spoilage organisms (SSOs) in Chinese sea bass, (ii) to develop stable FSG-SA active films with different concentrations of carvacrol, (iii) to investigate the effect of FSG-SA films containing carvacrol on the chemical, microbiological changes and organoleptic evaluation of Chinese sea bass during cold storage.

## 2. Materials and Methods

### 2.1. Evaluation of Antimicrobial Activity

#### 2.1.1. Determination of MIC and MBC of Carvacrol

The MICs of carvacrol for against *Vibrio parahemolyticus* (ATCC 17802), *Shewanella putrefaciens* (ATCC 49138), *Staphylococcus aureus* (ATCC 6538), and *Pseudomonas fluorescens* (ATCC 13525) were determined by the double dilution method as described by Cui et al. [23]. Different concentrations of carvacrol (98 % (GC), Aladdin Biochemical Technology Co., Ltd., Shanghai, China) were made as 0.03, 0.06, 0.125, 0.25, 0.50, 1.00, 2.00, 4.00 mg/L, respectively, with broth medium. All the strains were purchased from Nanjing Yiji Biochemical Technology co., LTD.

#### 2.1.2. The Effects of Carvacrol on the Bacterial Cell Membrane

The cell integrity of *V. Parahemolyticus, S. putrefaciens, S. aureus*, and *P. fluorescens* strains were examined by determining the release of nucleic acids and proteins into supernatant according to Xu et al. [24] and Cao et al. [25].

#### 2.1.3. Effects of Carvacrol on Bacterial Cell Wall

AKP activity was determined by the AKP detection kit (Jiancheng Biology Engineering Institute, Nanjing, China) using an ultraviolet spectrophotometer (Hitachi 3900, Hitachi Instruments, Japan) to detect the absorbance at 520 nm.

#### 2.1.4. Fourier Transform Infrared (FTIR)

FTIR spectra were analyzed in transmission mode by using Spotlight 400 FTIR spectrometer (PerkinElmer, USA) in the range of 400–4000 cm^−1^ at a resolution of 4 cm^−1^.

#### 2.1.5. Scanning Electron Microscopes

The tested bacterial cells were treated with 1 × MIC carvacrol for 3 h and the cells were collected by centrifugation at 10142× *g* at 4 °C for 15 min, washed three times with 0.01 M PBS (PH 7.4). Then the treatment of cells and observations of SEM was followed by the reported study [26] using a SEM system (MIRA3, Tescan, Czech Republic).

### 2.2. Properties of Active Films

#### 2.2.1. Preparation of the Active Films

The carvacrol/βCD (M.W. 1134.98, Aladdin Biochemical Technology Co., Ltd., Shanghai, China) emulsions were prepared using the way described by Sun et al. [27] and Shao et al. [28] with some modifications. Carvacrol (0.5 mL, 1.0 mL and 2.0 mL) and 5.0 g βCD were stirred mechanically in a beaker, respectively, and 4 g Tween 80 was added to make them homogeneously dispersed. Then 400 mL ultrapure water was added and stirred for 8 h at room temperature and ultrapure water was continue to add to a final emulsion volume of 1000 mL. The emulsion was obtained by continuous stirring for another 6 h. The corresponding concentrations of carvacrol in the carvacrol/βCD emulsions were 0.5, 1.0, 2.0 mL/L. Food grade flaxseed gum (FSG, 0.5 % *w*/*v*, Aladdin Biochemical Technology Co., Ltd., Shanghai, China), sodium alginate (SA, viscosity of 200 ± 20mpa.s, 1.5 % *w*/*v*, Aladdin Biochemical Technology Co., Ltd., Shanghai, China) and glycerol (1.5 % *v*/*w*) were dissolved in prepared carvacrol/βCD emulsions (1000 mL) at 40 °C and stirred for 4 h. Then, the mixture was treated with an Ultrasonic homogenizer (XEB-1000-P, Xiecheng Ultrasonic Equipment co. LTD, Shandong, China) at 20 KHz with the powder of 800 W for 10 min to obtain homogeneous coating solutions. Then they were removed the air bubbles by centrifugation at 11620× *g* for 15 min. The FSG-SA films containing carvacrol were prepared by casting 500 mL gelatinized suspensions on square glass plate (40 × 40 cm) and dried (at 25 °C, 50 % R.H.) in a constant temperature humidity chamber (KBF 240, Binder GmbH., Germany) for 36 h. The final active films were marked as FSG-SA, FSG-SA-0.5C, FSG-SA-1.0C, and FSG-SA-2.0C groups, respectively.

#### 2.2.2. Mechanical Properties

The TS and EB of prepared FSG-SA films containing carvacrol were measured using a TA-XT2i texture analyzer (Stable Micro Systems Ltd., Godalming, UK) according to Biao et al. [29].

#### 2.2.3. Antimicrobial Activity

The antimicrobial activities of the FSG-SA films containing carvacrol were carried out according to the method described by Aguilar-Sánchez et al. [30] and the results were expressed as the percentage of reduction in bacterial counts (Inhibition ratio, %).

#### 2.2.4. Antioxidant Activity

The antioxidant activities of FSG-SA films containing carvacrol were determined through DPPH radical scavenging assay, ABTS radical scavenging assay and Ferric reducing antioxidant power (FRAP) assay as proposed by Kaya et al. [31], Govindaswamy et al. [32], and Polumackanycz et al. [33], respectively.

#### 2.2.5. Differential Scanning Calorimetry (DSC) Determination

The analysis of the DSC patterns of the FSG-SA films containing carvacrol was conducted using DSC Q2000 (TA Instruments-Waters LLC, New Castle, DE, USA). The samples (about 13.5 mg) were hermetically encapsulated in aluminium pots heating from 50 to 250 °C at 10 °C/min under nitrogen protection.

### 2.3. Fish Storage Trial

#### 2.3.1. Preparation of Sea Bass Samples

Live Chinese sea bass (*Lateolabrax maculatus*) with an average weight of 1000 ± 50 g were purchased from a local aquatic product market in Luchao Port town (Shanghai, China). They were stunned with ice for 15 min and then killed using percussive stunning. The sea bass fillets, including skin, 150–160 g, were prepared and thoroughly washed with sterilized 1% NaCl solutions. The sea bass fillets samples were divided into five batches for (1) CK (uncoated), (2) FSG-SA (coated with FSG-SA without carvacrol), (3) FSG-SA-0.5C (coated with FSG-SA films containing 0.5 mL/L carvacrol), (4) FSG-SA-1.0C (coated with FSG-SA films containing 1.0 mL/L carvacrol), (5) FSG-SA-2.0C (coated with FSG-SA films containing 2.0 mL/L carvacrol). Different groups of sea bass fillets samples were coated with the corresponding freshly prepared active films and stored at −4.0 ± 0.1 °C in a refrigerator (BPS-250CB, Yiheng Thermostatic Chamber, Shanghai, China) for subsequent assessments at 3-day interval.

#### 2.3.2. Microbial Analysis

Representative 5 g portions of sea bass fillets samples were blended with 45 mL of sterilized normal saline (0.85 % NaCl) completely homogenized and then subjected to serial dilutions. The following microbiological analyses were performed [34]: (i) determination of TVC on plate count agar medium (Hope Bio-Technology Co., Ltd., Qingdao, China) with 0.4 mg/mL nystatin (Aladdin Biochemical Technology Co., Ltd., Shanghai, China) were incubated at 30 °C for 48 h, (ii) determination of H_2_S-producing bacteria on iron agar medium were incubated at 30 °C for 72 h, (iii) determination of *Pseudomonas* spp. on cetrimide agar medium were incubated at 30 °C for 72 h, (iv) determination of psychrophilic bacteria on plate count agar medium were incubated at 4 °C for 7 days.

#### 2.3.3. TVB-N Analysis

For TVB-N determinations, the steam distillation was performed with Kjeldahl analysis apparatus (Kjeltec8400, Foss, Denmark) as recommended by Guan et al. [4] and TVB-N values were expressed as mg N/100 g of sea bass fillets samples.

#### 2.3.4. K Value Analysis

The ATP-related compounds of sea bass fillets samples during cold storage were determined by a RP-HPLC procedure (Waters 2695, Milford, MA, USA) proposed by Shibata et al. [35] and K value was calculated as follows:

$$K\;\mathrm{value}\;(\%)=\frac{\mathrm{HxR}+\mathrm{Hx}}{\mathrm{ATP}+\mathrm{ADP}+\mathrm{AMP}+\mathrm{IMP}+\mathrm{HxR}+\mathrm{Hx}}\;\times 100\;$$

#### 2.3.5. Organoleptic Properties

The organoleptic properties of sea bass fillets samples were assessed with the method described by Miranda, et al. [36]. Ten experienced judges (four men and six women between 22 and 40 years old) received some training about the cold storage sea bass samples, focusing on color, flavor, morphology, and springiness using ten-grade marking system: 10.0–8.5 (excellent), 8.4–7.0 (good), 6.9–5.0 (fair), and 4.9–1.0 (rejectable).

### 2.4. Statistical Analysis

Experimental data were analyzed using SPSS 22.0 (IBM Corporation, Armonk, NY, USA). The one-way ANOVA procedure followed by Duncan’s multiple range tests was adopted to determine the significant difference (*p* < 0.05) between treatment means, and the results were expressed as means ± SD of three independent experiments.

## 3. Results and Discussion

### 3.1. Evaluation of Antimicrobial Activity

#### 3.1.1. The Minimum Inhibitory Concentration (MIC) and Minimum Bactericidal Concentration (MBC)

**Figure 1:**
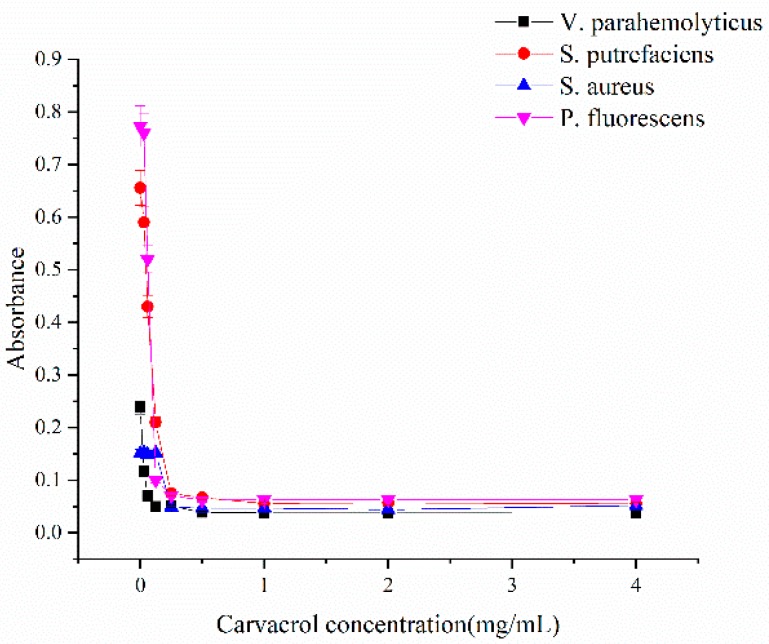
Inhibition of different concentrations of carvacrol on *V. parahemolyticus*, *S. putrefaciens*, *S. aureus* and *P. fluorescens.*

The antimicrobial effects of carvacrol against four common sea food-borne microorganisms were investigated by MIC and MBC. As shown in Figure 1, the MICs of carvacrol against *Vibrio Parahemolyticus*, *Shewanella putrefaciens*, *Staphylococcus aureus* and *Pseudomonas fluorescens* were 0.5, 0.5, 0.125, and 0.5 mg/mL, and the MBCs were 1.0, 1.0, 0.25, and 1.0 mg/mL, respectively, which were both lower than that of potassium sorbate and sodium benzoate [37]. Similarly, the MICs and MBCs of carvacrol against *Escherichia coli* O157: H7, *Salmonella Typhimurium, Listeria monocytogenes,* and *S. aureus* were found to be 0.5–1.0 mg/mL and 0.5–2.0 mg/mL, respectively [38]. The gram-positive bacterium (*S. aureus*) was more susceptible to carvacrol than that of gram-negative ones (*V. parahemolyticus*, *S. putrefaciens*, and *P. fluorescens*). Carvacrol could interact with lipophilic components of the cell membrane causing the permeability of H^+^ and K^+^, and finally destroy the essential functions to cell death [39]. At the same time, gram-negative bacteria possess a double membrane-containing cell membrane comparing to the single membrane structure of gram-positive bacteria [40]. In some researches, carvacrol was also recognized as more effective against gram-positive bacteria [41,42].

#### 3.1.2. Integrity of Cell Membrane

**Figure 2:**
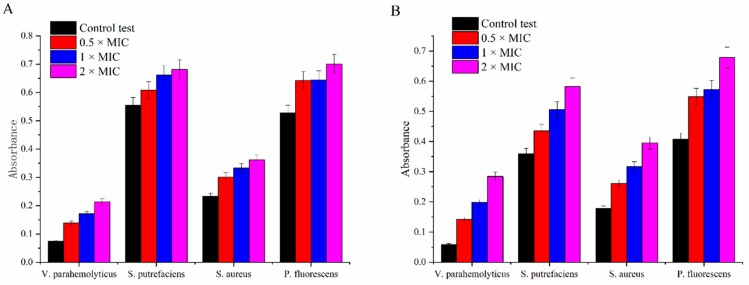
Release of intracellular nucleic acids (**A**) and proteins (**B**) from *V. parahemolyticus*, *S. putrefaciens*, *S. aureus*, and *P. fluorescens* respectively treated with carvacrol (control, 0.5 × MIC, 1 × MIC, 2 × MIC) for 3 h.

Once the integrity of the cell membrane is destroyed, the ions tend to flow out first and followed by macromolecular substances such as nucleic acids and proteins, which is a good indicator to evaluate the cell membrane integrity [40]. As shown in Figure 2, the absorbance at 260 and 280 nm increased with carvacrol concentration increasing for all tested bacterial strains, which illuminated the leakage of nucleic acids and proteins from strains into extracellular environment. The results were consistent with the previous researches, stating that carvacrol altered the membrane permeability resulting in the leakage of nucleic acids and proteins, which caused cell death [43,44].

#### 3.1.3. Results of Alkaline Phosphatase (AKP) Concentrations

**Figure 3:**
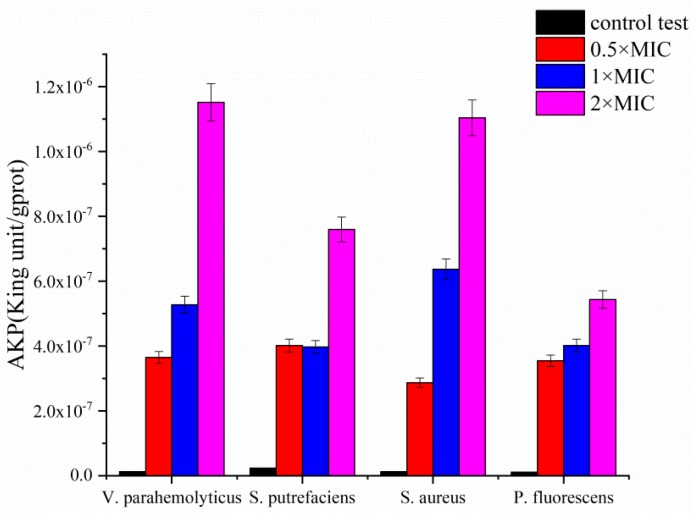
Effects on alkaline phosphatase (AKP) activities of *V. parahemolyticus*, *S. putrefaciens*, *S. aureus*, and *P. fluorescens* respectively treated with carvacrol (control, 0.5 × MIC, 1 × MIC, 2 × MIC) for 3 h.

AKP is an intracellular enzyme located between the cell wall and cell membrane, it could leak outside the bacteria into the extracellular environment when the cell wall was damaged [45]. As shown in Figure 3, without carvacrol, the OD_520_ of extracellular AKP were maintained at 1.7 × 10^−8^ King unit/gprot for all the tested bacteria strains. After treating with 1 × MIC carvacrol for 3 h, the OD_520_ values increased to 5.4, 3.7, 6.2, and 3.7 × 10^−7^ King unit/gprot for *V. parahemolyticus*, *S. putrefaciens*, *S. aureus*, and *P. fluorescens*, respectively. It was obvious that the AKP activities increased along with the increased carvacrol concentration from 0.5 × MIC to 2 × MIC for all the tested bacterial strains, which indicated carvacrol damage the cell wall resulting the leakage of AKP from the cells.

#### 3.1.4. FTIR Spectroscopy

**Figure 4:**
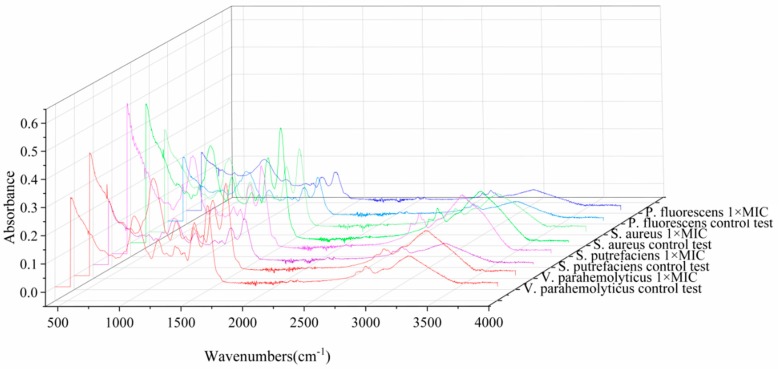
The fourier transform infrared (FTIR) spectroscopy of *V. parahemolyticus*, *S. putrefaciens*, *S. aureus*, and *P. fluorescens* respectively treated with carvacrol (control and 1 × MIC) for 3 h.

In order to testify if the carvacrol exhibited antibacterial activities against the tested bacterial strains, the secondary structures of biomacromolecule conjugate in strains were examined using the FTIR technique. For the tested bacterial strains, their characteristic absorption bands observed at around 3275 cm^−1^, 2928 cm^−1^, 1642 cm^−1^, 1537 cm^−1^, 1451 cm^−1^, 1378 cm^−1^, 1237 cm^−1^, and 1044 cm^−1^ were assigned to deformation of OH stretching vibration, CH stretching vibrations, CO stretching vibrations, proteinamide II, cellular structural protein, cellular structural protein, SO stretching vibration, and nucleic acid, respectively [46,47]. As shown in Figure 4, the absorption peaks of *V. parahemolyticus* at 2928cm^−1^, 1237cm^−1^ decreased, indicating that carvacrol destroys the phospholipid structure on the membrane. At the same time, the bands 1642 cm^−1^, 1537 cm^−1^, 1451 cm^−1^, and 1378 cm^−1^ also decreased, suggesting the proteins leaked out from strains into extracellular environment. The FTIR spectroscopy of *S. putrefaciens* was similar with that of *V. parahemolyticus*, however, the absorption peaks at 1065 cm^−1^ decreased indicting the nucleic acid leakage resulted in the growth inhibition of *S. putrefaciens*. For *S. aureus*, the absorption peaks at 2920 cm^−1^, indicating that carvacrol destroys the phospholipid structure on the membrane. Other absorption peaks did not have significant difference. For *P. fluorescens*, the absorption peaks at 1522 cm^−1^, 1451 cm^−1^, and 1383 cm^−1^ increased, which may due to some changes produced in the secondary structure of the protein. However, the bands at 3280 cm^−1^, 2920 cm^−1^, and 1227 cm^−1^ increased indicting the phospholipid content increased and carvacrol did not show strong inhibitory effect to *P. fluorescens.*

#### 3.1.5. Morphological Changes

**Figure 5:**
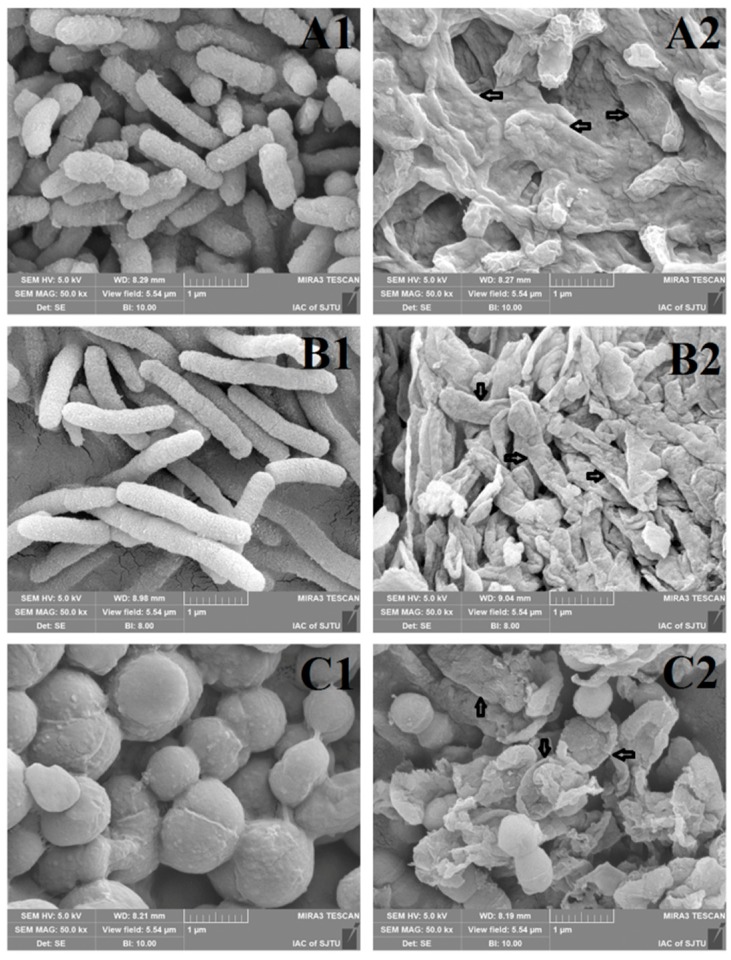

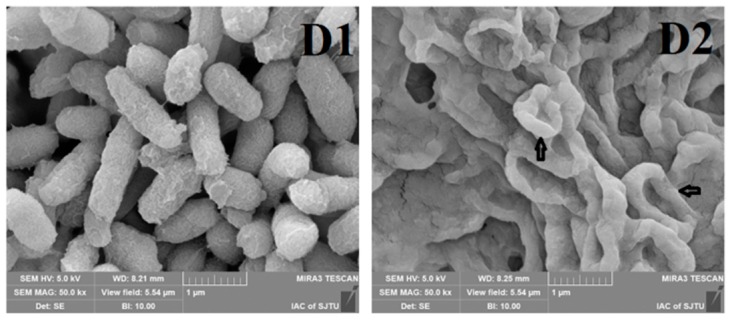
Scanning electron microscope images of *V. parahemolyticus* (**A**), *S. putrefaciens* (**B**), *S. aureus* (**C**), and *P. fluorescens* (**D**) (A1–D1. Untreated, A2–D2. addition of 1 × MIC carvacrol for 3 h).

The morphological properties of *V. parahemolyticus*, *S. putrefaciens*, *S. aureus*, and *P. fluorescens* without/with carvacrol exposure were observed by SEM. The SEM images showed that the untreated bacterial cells (Figure 5A1–D1) displayed regular morphology, with a smooth and intact surface without releasing significant intracellular components and cell surface ruptures or pores. However, bacterial cells treated with 1 × MIC carvacrol for 3 h (Figure 5A2–D2) were seriously damaged. As shown in Figure 5A2 and 3C2, *V. parahemolyticus* and *S. putrefaciens* treated with carvacrol showed irregular wrinkles, destruction, and adhesion. Figure 5D2 stated that *P. fluorescens* cells treated with carvacrol showed an irregular wrinkles and central dip on its surface. However, *S. aureus* treated with carvacrol showed irregular wrinkles and destruction for most of the cells, some cells were still intact due to lipopolysaccharide. Overall, the morphological results indicated that the all the tested bacterial cells treated with 1 × MIC carvacrol for 3 h could cause the external structure damaged and leaking of cytoplasmic components [48].

### 3.2. Edible Films Properties

#### 3.2.1. Mechanical Properties

Differences in tensile properties of FSG-SA films with carvacrol incorporated at different concentrations were evaluated. TS and EB, representing for film strength and flexibility, are used for determining the mechanical properties of active films and could be related with chemical structure [49]. As shown in Table 1, the TS and EB of FSG-SA film is 58.70 MPa and 4.90 %, respectively, and the addition of carvacrol into FSG-SA films caused significant (*p* < 0.05) decreases in TS and EB. The incorporation of carvacrol in FSG-SA films could result in the structural discontinuities, which accounted for the lowest resistance to film strength and flexibility of FSG-SA films. The effectiveness of carvacrol is most likely to be due to the hydrophobic group. Some researchers proposed that the addition of hydrophobic agents into active films could result in the development of structural discontinuities with less flexibility and resistance to fracture [50,51]. Similar results were also observed when carvacrol was added into isolated soy protein [52], chitosan films [53].

#### 3.2.2. Antimicrobial Activities of Films

**Figure 6:**
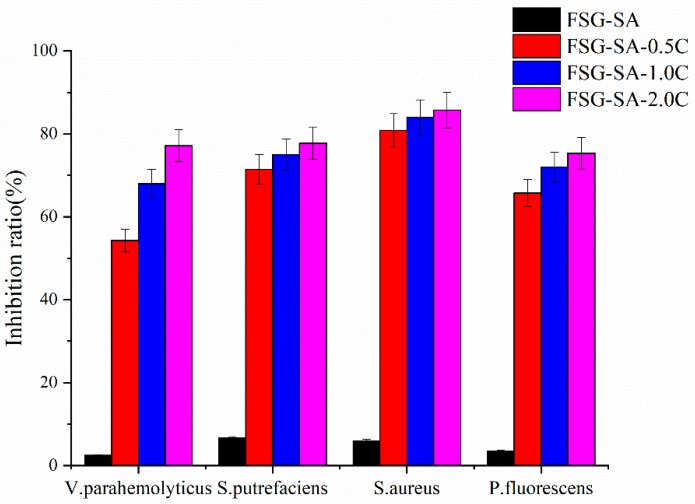
Inhibition effect of flaxseed gum-sodium alginate active films containing carvacrol (0, 0.5, 1.0, 2.0 mL/L, respectively) on *V. parahemolyticus*, *S. putrefaciens*, *S. aureus*, and *P. fluorescens*.

Figure 6 shows the antimicrobial activities exhibited by the inhibition ratio of FSG-SA films containing carvacrol against gram-negative bacteria (*V. parahemolyticus*, *S. putrefaciens*, and *P. fluorescens*) and gram-positive one (*S. aureus*). As shown in Figure 6, FSG-SA films containing carvacrol exhibited good antimicrobial activity against the tested bacteria and the inhibition ratio increased with the increased carvacrol concentrations (*p* < 0.05). However, the FSG-SA films presented different inhibition ratio depending on the sensitivity of the tested strains to carvacrol. Therefore, *V. parahemolyticus* was the most resistant strain to FSG-SA films containing carvacrol and *S. aureus* was the most sensitive, which were also supported by the MIC and MBC results of carvacrol.

#### 3.2.3. Antioxidant Activities of Films

**Figure 7:**
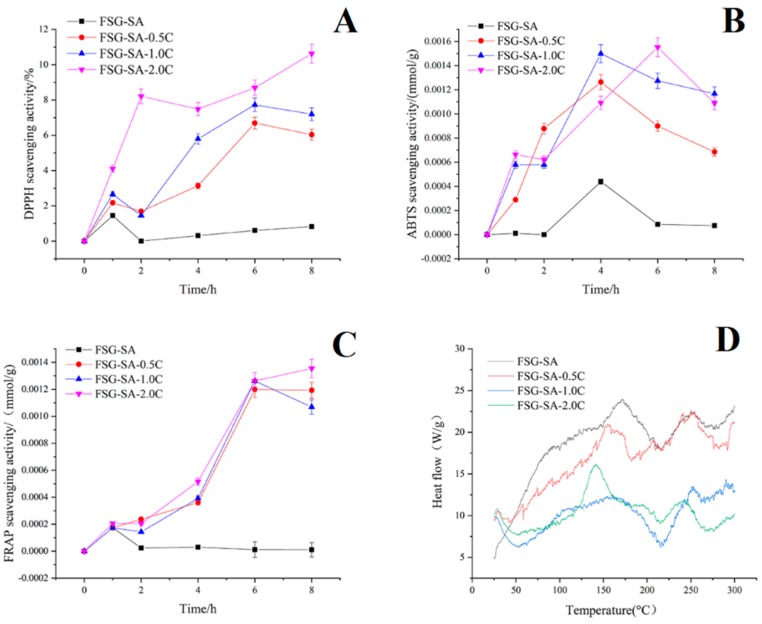
DPPH radical scavenging assay (**A**), ABTS radical scavenging assay (**B**), and Ferric reducing antioxidant power assay (**C**) and differential scanning calorimetry results (**D**) of flaxseed gum-sodium alginate active films containing carvacrol (0, 0.5, 1.0, 2.0 mL/L, respectively).

Antioxidant packaging is an important category of active packaging and very promising for prolonging the shelf life of food [53]. The antioxidant activities of FSG-SA films containing carvacrol as monitored by DPPH, ABTS, and FRAP were investigated. As shown in Figure 7, the antioxidant activities determined by DPPH, ABTS, and FRAP showed dose-dependent increment (*p* < 0.05) and FSG-SA-2.0C exhibited the highest antioxidant activity (Figure 7A–C). The antioxidant activity was only found in the films contained carvacrol, while the FSG-SA had a negligible antioxidant activity. The antioxidant capacity of carvacrol relies on the steric and electronic effect of its ring and the presence of the hydroxyl group, which could provide hydrogen atoms [54]. Similar results were previously reported in fish gelatin films enriched with carvacrol [54], chitosan films containing carvacrol [43], and so on.

#### 3.2.4. DSC Analysis

DSC was implemented to measure the glass transition temperature (Tg) of FSG-SA films containing carvacrol and Tg is closer to the compatibility between FSG-SA based film and carvacrol. [55,56]. As shown in Figure 7D, The Tg of FSG-SA-0.5C, FSG-SA-1.0C, and FSG-SA-2.0C was lower than that of FSG-SA, which potentially resulted from the low interaction between the OH groups of FSG-SA chain with OH, CO, or COOH chemical group of carvacrol via hydrogen bonding [57]. Yahyaoui et al. also reported that carvacrol added into the polylactic acid films led to a low decline in crystallization temperature [58].

### 3.3. Fish Storage Trial

#### 3.3.1. Microbiological Analyses

**Figure 8:**
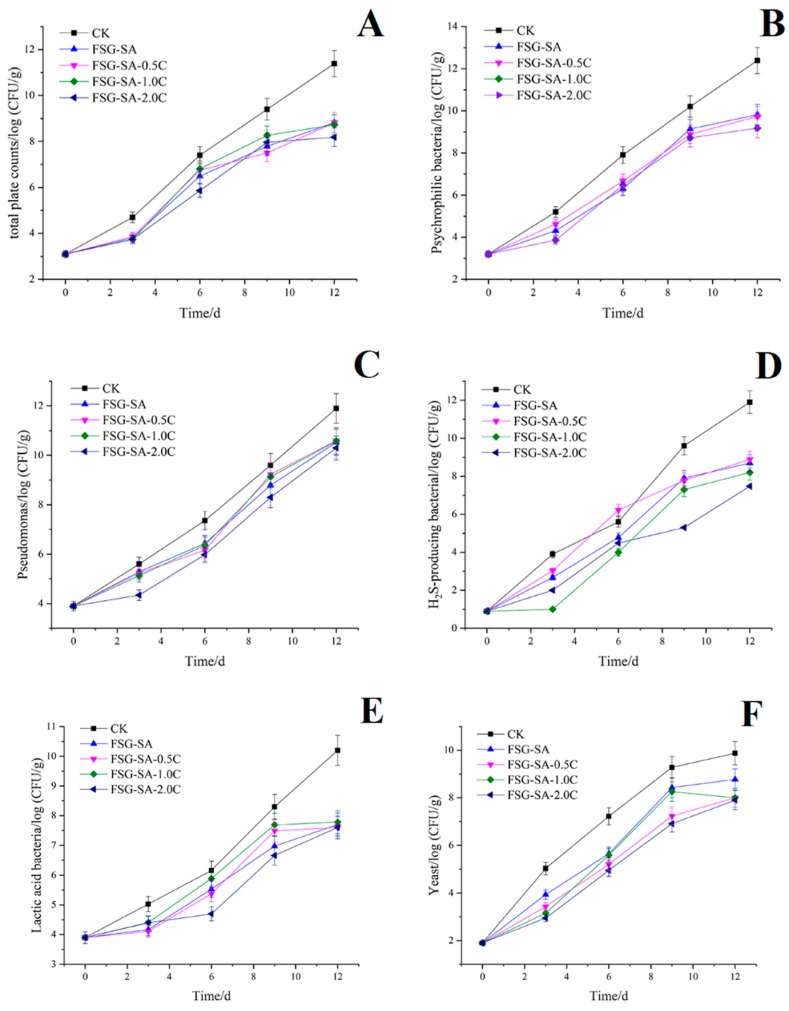
Changes in total viable count (**A**), Psychrophilic bacteria (**B**), *Pseudomonas* (**C**), H_2_S-producing bacteria (**D**), lactic acid bacteria (**E**) and yeast-mould (**F**) counts of Chinese sea bass fillets samples during cold storage at 4 °C (CK: uncoated, FSG-SA: coated with FSG-SA without carvacrol, FSG-SA-0.5C: coated with FSG-SA films containing 0.5 mL/L carvacrol, FSG-SA-1.0C: coated with FSG-SA films containing 1.0 mL/L carvacrol, FSG-SA-2.0C: coated with FSG-SA films containing 2.0 mL/L carvacrol).

Figure 8 shows the counts corresponding to the growth of the total viable counts (TVC), Psychrophilic bacteria, *Pseudomonas*, H_2_S-producing bacteria, lactic acid bacteria, and yeast-mould counts of sea bass fillets samples during refrigerated storage. The initial mesophiles count was about 3.1 log CFU/g (Figure 8A), which was at low microbial level for the starting sea bass fillets samples. The mesophiles number increased during storage for all sea bass fillets samples and the FSG-SA-2.0C samples had significantly lower mesophiles number than other samples during whole storage period. At day 6, CK and FSG-SA samples should be removed due to exceeding the allowed maximum limit of 7.0 log CFU/g. Carvacrol could prolong the shelf life of sea bass fillets samples because of its remarkable inhibitory effect and retard TVC growth [59], similar results also shown towards the increase of Psychrophilic bacteria, *Pseudomonas*, H_2_S-producing bacteria, lactic acid bacteria, and yeast-mould. Psychrotrophic bacteria could cause deterioration in odor, texture and flavor in sea bass fillets samples through the production of metabolic compounds such as biogenic amines, volatile sulfides, aldehydes and ketones [60]. At day 6, the total psychrophilic bacteria counts for CK, FSG-SA, FSG-SA-0.5C, FSG-SA-1.0C, and FSG-SA-2.0C samples were 7.8, 5.9, 6.2, 6.0, and 6.1 log CFU/g (Figure 8B), respectively, indicating that the quality of sea bass fillets samples was preserved by carvacrol addition. *Pseudomonas* spp. had a similar growth pattern with that of mesophilic microbes (Figure 8C), which suggests that the aerobic spoilage bacteria dominated for sea bass fillets samples during cold storage. H_2_S-producing bacteria is one of the specific spoilage organisms in sea bass during cold storage [61]. An initial count of H_2_S-producing bacteria in sea bass fillets samples was approximately 1.1 log CFU/g (Figure 8D). At the end of storage, samples treated with carvacrol presented lower counts. LAB could produce organic acids and ethanol in sea bass fillets samples during cold storage and the initial count was approximately 3.7 log CFU/g (Figure 8E). However, after 9 days of storage, the LAB counts reached to 8.3, 7.0, 6.7 7.6, and 6.5 log CFU/g for CK, FSG-SA, FSG-SA-0.5C, FSG-SA-1.0C, and FSG-SA-2.0C, respectively. The yeast-mould counts of sea bass fillets samples at day 0 were counted as 1.8 log CFU/g (Figure 8F). After 6 days of storage, yeast-mould counts 7.3, 5.4, 5.0, 5.3 and 4.7 log CFU/g for CK, FSG-SA, FSG-SA-0.5C, FSG-SA-1.0C, and FSG-SA-2.0C, respectively.

#### 3.3.2. Total Volatile Base Nitrogen (TVB-N)

**Figure 9:**
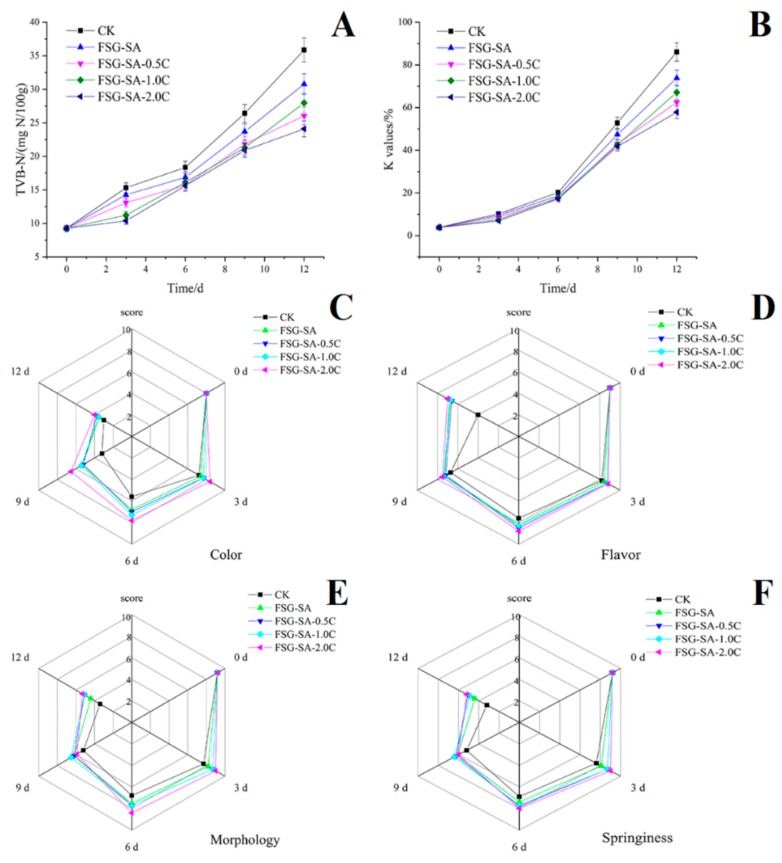
Changes in total volatile basic nitrogen (TVB-N, **A**), K values (**B**), organoleptic properties results (color: **C**, flavor: **D**, morphology: **E**, springiness: **F**) of Chinese sea bass fillets samples during cold storage at 4 °C (CK: uncoated, FSG-SA: coated with FSG-SA without carvacrol, FSG-SA-0.5C: coated with FSG-SA films containing 0.5 mL/L carvacrol, FSG-SA-1.0C: coated with FSG-SA films containing 1.0 mL/L carvacrol, FSG-SA-2.0C: coated with FSG-SA films containing 2.0 mL/L carvacrol).

The TVB-N value of fresh sea bass fillets sample was 8.34 mg/100 g fish muscle (Figure 9A) indicating quite freshness of sea bass fillet samples at the beginning. TVB-N values slowly increased at the beginning and had a sharp increase from day 6. The increase TVB-N formation was especially obvious in CK and FSG-SA sea bass fillets samples, however, the samples with FSG-SA films containing carvacrol significantly reduced the formation of TVB-N during storage comparing with the CK and FSG-SA samples. The TVB-N values of CK, FSG-SA, FSG-SA-0.5C, FSG-SA-1.0C, and FSG-SA-2.0C sea bass fillets samples reached to 35.88, 30.78, 26.04, 27.98, and 24.09 mg N/100 g fish muscle, respectively, at the end of storage, which signified sea bass fillets samples treated with FSG-SA films containing carvacrol did not exceed the upper limit (30 mg N/100 g) throughout cold storage [62]. High TVB-N values in aquatic products preservation indicates that nitrogen-containing substances accumulated, which may due to proteolytic bacteria decomposed protein and nucleic acid [63].

#### 3.3.3. K Value

Changes in K value of sea bass fillets samples during cold storage are presented in Figure 9B. The K value of fresh sea bass samples was 6.21 % and increased during cold storage. The unacceptable limit for K value is 60% [64] and the CK, FSG-SA, and FSG-SA-0.5C samples exceeded the maximum permissible level at the end of storage. The usage of FSG-SA films containing carvacrol significantly retarded the K-values increase during storage, which had an analogous trend with the TVB-N values. These results indicated that FSG-SA coatings containing carvacrol could suppress the degradation of ATP resulting from spoilage organism and microbial enzymes and keep good quality of sea bass fillets samples during cold storage.

#### 3.3.4. Organoleptic Evaluation Results

The acceptability of sea bass fillets samples during cold storage depends upon their changes in organoleptic characteristics. Figure 9C–9F display the organoleptic evaluation results for the sea bass fillets samples including color, flavor, morphology, and springiness during cold storage. At the beginning, all sea bass fillets samples had high organoleptic scores and they were of excellent quality, and then a significant quality loss in all sea bass fillets samples was observed (*p* < 0.05) during cold storage. However, sea bass fillets samples treated with FSG-SA films containing carvacrol had significant higher organoleptic scores than those of the CK and FSG-SA samples (*p* < 0.05). Sea bass fillets samples treated with higher carvacrol concentration (1.0 and 2.0 mL/L) consistently proved better organoleptic characteristics and shelf life than other samples. The CK sea bass fillets samples were considered unacceptable by the panelists on day 9, when the samples were spoiled with off-odor and loose elasticity. No significant difference (*p* > 0.05) was detected among sea bass fillets samples treated with FSG-SA-1.0C and FSG-SA-2.0C films on day 12. However, both of them were not suitable for consumption suggested by panelists. Previous studies have revealed excellent relevance among microbial and chemical qualities with organoleptic characteristics [65], which were consistent with our research.

## 4. Conclusions

The present study showed that carvacrol has good antibacterial activity against *V.* *parahemolyticus*, *S.* *putrefaciens*, *S.* *aureus* and *P. fluorescens*. The cell integrity results demonstrated that carvacrol could damage cell membrane, eventually leading to cell death. SEM results further confirmed the disruption of cell membrane caused by carvacrol. FSG-SA films containing carvacrol presented better antimicrobial and antioxidant activities against the tested bacteria studied, however, they had less TS and EB compared to the control film. Sea bass fillets samples coated with FSG-SA containing carvacrol maintained chemical qualities and kept excellent organoleptic characteristics during cold storage, which mainly due to that carvacrol could effectively suppress the growth of spoilage microorganisms. 1.0 or 2.0 mg/L carvacrol additions had similar effects in slowing down sea bass fillets samples spoilage, however, 2.0 mg/L carvacrol addition gave the FSG-SA film strong flavor. Therefore, 1.0 mg/L carvacrol addition could be suitable for maintaining the freshness of sea bass fillets samples and also respond the principle of using as little food additive as possible. 

## Figures and Tables

**Table 1:** Mechanical properties of flaxseed gum-sodium alginate active films.

Film Sample	Tensile Strength (MPa)	Elongation at Break (%)
FSG-SA	58.70 ± 0.53 ^a^	4.90 ± 0.13 ^a^
FSG-SA-0.5C	41.34 ± 0.27 ^c^	4.41 ± 0.10 ^ab^
FSG-SA-1.0C	44.95 ± 0.63 ^b^	4.05 ± 0.08 ^ab^
FSG-SA-2.0C	40.52 ± 0.37 ^c^	3.83 ± 0.16 ^b^

## References

[1] Zhou Q., Li P., Fang S., Liu W., Mei J., Xie J. (2019). Preservative effects of gelatin active coating enriched with eugenol emulsion on Chinese seabass (*Lateolabrax maculatus*) during superchilling (−0.9 °C) storage. Coatings.

[2] Parlapani F.F., Haroutounian S.A., Nychas G.-J.E., Boziaris I.S. (2015). Microbiological spoilage and volatiles production of gutted European sea bass stored under air and commercial modified atmosphere package at 2 °C. Food Microbiol..

[3] Volpe M.G., Coccia E., Siano F., Di Stasio M., Paolucci M. (2019). Rapid Evaluation Methods for Quality of Trout (*Oncorhynchus mykiss*) Fresh Fillet Preserved in an Active Edible Coating. Foods.

[4] Guan W., Ren X., Li Y., Mao L. (2019). The beneficial effects of grape seed, sage and oregano extracts on the quality and volatile flavor component of hairtail fish balls during cold storage at 4 °C. LWT Food Sci. Technol..

[5] Álvarez A., Fontanillas R., García-García B., Hernández M.D. (2018). Impact of Dietary Oil Source on the Shelf-Life of Gilthead Seabream (*Sparus aurata*). J. Aquat. Food Prod. Technol..

[6] Dou L., Li B., Zhang K., Chu X., Hou H. (2018). Physical Properties and Antioxidant Activity of Gelatin-Sodium Alginate Edible Films with Tea Polyphenol. Int. J. Biol. Macromol..

[7] Shahbazi Y., Shavisi N. (2019). Effects of Sodium Alginate Coating Containing Mentha Spicata Essential Oil and Cellulose Nanoparticles on Extending the Shelf Life of Raw Silver Carp (*Hypophthalmichthys molitrix*) fillets. Food Sci. Biotechnol..

[8] Nie X., Wang L., Wang Q., Lei J., Hong W., Huang B., Zhang C. (2018). Effect of a Sodium Alginate Coating Infused with Tea Polyphenols on the Quality of Fresh Japanese Sea Bass (Lateolabrax japonicas) Fillets. J. Food Sci..

[9] Vital A.C.P., Guerrero A., Ornaghi M.G., Kempinski E.M.B.C., Sary C., Monteschio J.d.O., Matumoto-Pintro P.T., Ribeiro R.P., do Prado I.N. (2018). Quality and Sensory Acceptability of Fish Fillet (*Oreochromis niloticus*) with Alginate-Based Coating Containing Essential Oils. J. Food Sci. Technol..

[10] Cheng M., Wang J., Zhang R., Kong R., Lu W., Wang X. Characterization and application of the microencapsulated carvacrol/sodium alginate films as food packaging materials. Int. J. Biol. Macromol..

[11] Zhang S., Wei F., Han X. (2018). An edible film of sodium alginate/pullulan incorporated with capsaicin. New J. Chem..

[12] Pires A.M.T., Shirai M.A., Grossmann M.V.E., Yamashita F. (2013). Active biodegradable packaging for fresh pasta. LWT Food Sci. Technol..

[13] Neira L.M., Agustinelli S.P., Ruseckaite R.A., Martucci J.F. (2019). Shelf Life Extension of Refrigerated Breaded Hake Medallions Packed into Active Edible Fish Gelatin Films. Packag. Technol. Sci..

[14] Alves V.L.C.D., Rico B.P.M., Cruz R.M.S., Vicente A.A., Khmelinskii I., Vieira M.C. (2018). Preparation and Characterization of a Chitosan Film with Grape Seed Extract-Carvacrol Microcapsules and its Effect on the Shelf-Life of Refrigerated Salmon (*Salmo salar*). LWT Food Sci. Technol..

[15] Chaparrohernández S., Ruízcruz S., Márquezríos E., Ocañohiguera V.M., Valenzuelalópez C.C., Ornelaspaz J.D.J., Deltorosánchez C.L., Chaparrohernández S., Ruízcruz S., Márquezríos E. (2015). Effect of Chitosan-Carvacrol Edible Coatings on the Quality and Shelf Life of Tilapia (*Oreochromis niloticus*) Fillets Stored in Ice. Food Sci. Technol..

[16] Marchese A., Arciola C.R., Coppo E., Barbieri R., Barreca D., Chebaibi S., Sobarzo-Sánchez E., Nabavi S.F., Nabavi S.M., Daglia M. (2018). The Natural Plant Compound Carvacrol as an Antimicrobial and Anti-biofilm Agent: Mechanisms, Synergies and Bio-inspired Anti-infective Materials. Biofouling.

[17] Nash J.J., Erk K.A. (2017). Stability and Interfacial Viscoelasticity of Oil-water Nanoemulsions Stabilized by Soy Lecithin and Tween 20 for the Encapsulation of Bioactive Carvacrol. Colloid. Surf. A..

[18] Kamdem D.P., Shen Z., Nabinejad O. (2019). Development of Biodegradable Composite Chitosan-based Films Incorporated with Xylan and Carvacrol for Food Packaging Application. Food Packag. Shelf.

[19] Kurkov S.V., Loftsson T. (2013). Cyclodextrins. Int. J. Pharm..

[20] Szente L., Fenyvesi É. (2017). Cyclodextrin-lipid Complexes: Cavity Size Matters. Struct. Chem..

[21] Kfoury M., Auezova L., Greige-Gerges H., Fourmentin S. (2015). Promising Applications of Cyclodextrins in Food: Improvement of Essential Oils Retention, Controlled Release and Antiradical Activity. Carbohyd. Polym..

[22] López-Nicolás J.M., García-Carmona F. (2008). Rapid, Simple and Sensitive Determination of the Apparent Formation Constants of Trans-resveratrol Complexes with Natural Cyclodextrins in Aqueous Medium Using HPLC. Food Chem..

[23] Cui H., Zhang C., Li C., Lin L. (2018). Antimicrobial Mechanism of Clove Oil on *Listeria monocytogenes*. Food Control.

[24] Xu J.-G., Liu T., Hu Q.-P., Cao X.-M. (2016). Chemical Composition, Antibacterial Properties and Mechanism of Action of Essential Oil from Clove Buds against *Staphylococcus aureus*. Molecules.

[25] Cao J., Fu H., Gao L., Zheng Y. (2019). Antibacterial Activity and Mechanism of Lactobionic Acid Against *Staphylococcus aureus*. Folia Microbiol. (Praha).

[26] Guan X., Ge D., Li S., Huang K., Liu J., Li F. (2019). Chemical Composition and Antimicrobial Activities of Artemisia argyi Lévl. et Vant Essential Oils Extracted by Simultaneous Distillation-Extraction, Subcritical Extraction and Hydrodistillation. Molecules.

[27] Xinyu S., Xiaoban G., Mingyu J., Jiulin W., Wenjin Z., Jianhua W., Cui C., Li C., Qiqing Z. (2019). Preservative Effects of Fish Gelatin Coating Enriched with CUR/βCD Emulsion on Grass Carp (*Ctenopharyngodon idellus*) Fillets during Storage at 4 °C. Food Chem..

[28] Shao Y., Wu C., Wu T., Li Y., Chen S., Yuan C., Hu Y. (2018). Eugenol-chitosan Nanoemulsions by Ultrasound-mediated Emulsification: Formulation, Characterization and Antimicrobial Activity. Carbohyd. Polym..

[29] Biao Y., Yuxuan C., Qi T., Ziqi Y., Yourong Z., McClements D.J., Chongjiang C. (2019). Enhanced Performance and Functionality of Active Edible Films by Incorporating Tea Polyphenols into Thin Calcium Alginate Hydrogels. Food Hydrocolloid..

[30] Aguilar-Sánchez R., Munguía-Pérez R., Reyes-Jurado F., Navarro-Cruz A.R., Cid-Pérez T.S., Hernández-Carranza P., Beristain-Bauza S.d.C., Ochoa-Velasco C.E., Avila-Sosa R. (2019). Structural, Physical, and Antifungal Characterization of Starch Edible Films Added with Nanocomposites and Mexican Oregano (*Lippia berlandieri* Schauer) Essential Oil. Molecules.

[31] Kaya M., Ravikumar P., Ilk S., Mujtaba M., Akyuz L., Labidi J., Salaberria A.M., Cakmak Y.S., Erkul S.K. (2018). Production and Characterization of Chitosan Based Edible Films from Berberis crataegina’s Fruit Extract and Seed Oil. Innov. Food Sci. Emerg. Technol..

[32] Govindaswamy R., Robinson J.S., Geevaretnam J., Pandurengan P. (2018). Physico-functional and Anti-oxidative Properties of Carp Swim Bladder Gelatin and Brown Seaweed Fucoidan Based Edible Films. J. Packag. Technol. Res..

[33] Polumackanycz M., Sledzinski T., Goyke E., Wesolowski M., Viapiana A. (2019). A Comparative Study on the Phenolic Composition and Biological Activities of *Morus alba* L. Commercial Samples. Molecules.

[34] Rollini M., Nielsen T., Musatti A., Limbo S., Piergiovanni L., Hernandez Munoz P., Gavara R. (2016). Antimicrobial Performance of Two Different Packaging Materials on the Microbiological Quality of Fresh Salmon. Coatings.

[35] Shibata M., ElMasry G., Moriya K., Rahman M.M., Miyamoto Y., Ito K., Nakazawa N., Nakauchi S., Okazaki E. (2018). Smart Technique for Accurate Monitoring of ATP Content in Frozen Fish Fillets using Fluorescence Fingerprint. LWT Food Sci. Technol..

[36] Miranda J.M., Carrera M., Barros-Velázquez J., Aubourg S.P. (2018). Impact of Previous Active Dipping in *Fucus spiralis* Extract on the Quality Enhancement of Chilled Lean Fish. Food Control.

[37] Zhang S., Xiong J., Lou W., Ning Z., Zhang D., Yang J. (2019). Antimicrobial Activity and Action Mechanism of Triglycerol Monolaurate on Common Foodborne Pathogens. Food Control.

[38] Rivas L., Mcdonnell M.J., Burgess C.M., O’Brien M., Navarro-Villa A., Fanning S., Duffy G. (2010). Inhibition of Verocytotoxigenic *Escherichia coli* in Model Broth and Rumen Systems by Carvacrol and Thymol. Int. J. Food Microbiol..

[39] Ultee A., Bennik M.H.J., Moezelaar R. (2002). The Phenolic Hydroxyl Group of Carvacrol is Essential for Action Against the Food-borne Pathogen *Bacillus cereus*. Appl. Environ. Microbiol..

[40] Zhang Y., Liu X., Wang Y., Jiang P., Quek S.Y. (2016). Antibacterial Activity and Mechanism of Cinnamon Essential Oil Against *Escherichia coli* and *Staphylococcus aureus*. Food Control.

[41] Sung S.Y., Sin L.T., Tee T.T., Bee S.T., Rahmat A.R., Rahman W.A.W.A., Tan A.C., Vikhraman M. (2013). Antimicrobial Agents for Food Packaging Applications. Trends Food Sci. Technol..

[42] Fernández-Pan I., Maté J.I., Gardrat C., Coma V. (2015). Effect of Chitosan Molecular Weight on the Antimicrobial Activity and Release Rate of Carvacrol-enriched Films. Food Hydrocoll..

[43] Nostro A., Papalia T. (2012). Antimicrobial Activity of Carvacrol: Current Progress and Future Prospectives. Recent Pat. Anti-Infe. Drug Discov..

[44] Guarda A., Rubilar J.F., Miltz J., Galotto M.J. (2011). The Antimicrobial Activity of Microencapsulated Thymol and Carvacrol. Int. J. Food Microbiol..

[45] Tang H., Chen W., Dou Z.-M., Chen R., Hu Y., Chen W., Chen H. (2017). Antimicrobial Effect of Black Pepper Petroleum Ether Extract for the Morphology of *Listeria monocytogenes* and *Salmonella typhimurium*. J. Food Sci. Technol..

[46] Liang C., Yuan F., Liu F., Wang Y., Gao Y. (2014). Structure and Antimicrobial Mechanism of ɛ-Polylysine-Chitosan Conjugates through Maillard Reaction. Int. J. Biol. Macromol..

[47] Liu M., Liu Y., Cao M.-J., Liu G.-M., Chen Q., Sun L., Chen H. (2017). Antibacterial Activity and Mechanisms of Depolymerized Fucoidans Isolated from *Laminaria japonica*. Carbohyd. Polym..

[48] Chen M., Zhao Z., Meng H., Yu S. (2017). The Antibiotic Activity and Mechanisms of Sugar Beet (*Beta vulgaris*) Molasses Polyphenols Against Selected Food-borne Pathogens. LWT Food Sci. Technol..

[49] Zavareze E.D.R., Vania Zanella P., Bruna K., El Halal S.L.M., Moacir Cardoso E., Carlos P.H., Dias A.R.G. (2012). Development of Oxidised and Heat-moisture Treated Potato Starch Film. Food Chem..

[50] Gaofeng Y., Hua L., Bingjie Y., Xiaoe C., Haiyan S. (2015). Physical Properties, Antioxidant and Antimicrobial Activity of Chitosan Films Containing Carvacrol and Pomegranate Peel Extract. Molecules.

[51] Rubilar J.F., Rui M.S.C., Silva H.D., Vicente A.A., Khmelinskii I., Vieira M.C. (2013). Physico-mechanical Properties of Chitosan Films with Carvacrol and Grape Seed Extract. J. Food Eng..

[52] Otoni C.G., Avena-Bustillos R.J., Olsen C.W., Bilbao-Sáinz C., McHugh T.H. (2016). Mechanical and Water Barrier Properties of Isolated Soy Protein Composite Edible Films as Affected by Carvacrol and Cinnamaldehyde Micro and Nanoemulsions. Food Hydrocolloid..

[53] Moradi M., Tajik H., Rohani S.M.R., Mahmoudian A. (2016). Antioxidant and Antimicrobial Effects of Zein Edible Film Impregnated with *Zataria multiflora* Boiss. Essential Oil and Monolaurin. LWT Food Sci. Technol..

[54] Neira L.M., Martucci J.F., Stejskal N., Ruseckaite R.A. (2019). Time-dependent Evolution of Properties of Fish Gelatin Edible Films Enriched with Carvacrol during Storage. Food Hydrocolloid..

[55] Hosseini S.F., Rezaei M., Zandi M., Farahmandghavi F. (2016). Preparation and Characterization of Chitosan Nanoparticles-Loaded Fish Gelatin-Based Edible Films. J.Food Process Eng..

[56] Park S.Y., Kim J.Y., Youn H.J., Choi J.W. (2017). Fractionation of Lignin Macromolecules by Sequential Organic Solvents Systems and their Characterization for Further Valuable Applications. Int. J. Biol. Macromol..

[57] Muriel-Galet V., Cran M.J., Bigger S.W., Hernández-Muñoz P., Gavara R. (2015). Antioxidant and Antimicrobial Properties of Ethylene Vinyl Alcohol Copolymer Films Based on the Release of Oregano Essential Oil and Green Tea Extract Components. J. Food Eng..

[58] Yahyaoui M., Gordobil O., Herrera Díaz R., Abderrabba M., Labidi J. (2016). Development of Novel Antimicrobial Films Based on Poly(lactic acid) and Essential Oils. React. Funct. Polym..

[59] Singh S., Lee M., Gaikwad K.K., Lee Y.S. (2018). Antibacterial and Amine Scavenging Properties of Silver–silica Composite for Post-harvest Storage of Fresh Fish. Food Bioprod. Process..

[60] Vatavali K., Karakosta L., Nathanailides C., Georgantelis D., Kontominas M.G. (2013). Combined Effect of Chitosan and Oregano Essential Oil Dip on the Microbiological, Chemical, and Sensory Attributes of Red Porgy (*Pagrus pagrus*) Stored in Ice. Food Bioprocess Technol..

[61] Cheng J.H., Sun D.W., Wei Q. (2017). Enhancing Visible and Near-Infrared Hyperspectral Imaging Prediction of TVB-N Level for Fish Fillet Freshness Evaluation by Filtering Optimal Variables. Food Anal. Method..

[62] Volpe M.G., Siano F., Paolucci M., Sacco A., Sorrentino A., Malinconico M., Varricchio E. (2015). Active Edible Coating Effectiveness in Shelf-life Enhancement of Trout (*Oncorhynchusmykiss*) Fillets. LWT Food Sci. Technol..

[63] Cheng J.-H., Sun D.-W., Qu J.-H., Pu H.-B., Zhang X.-C., Song Z., Chen X., Zhang H. (2016). Developing a Multispectral Imaging for Simultaneous Prediction of Freshness Indicators during Chemical Spoilage of Grass Carp Fish Fillet. J. Food Eng..

[64] Lan W., Che X., Xu Q., Wang T., Du R., Xie J., Hou M., Lei H. (2018). Sensory and Chemical Assessment of Silver Pomfret (*Pampus argenteus*) Treated with *Ginkgo biloba* Leaf Extract Treatment during Storage in Ice. Aquac. Fish..

[65] Silbande A., Adenet S., Chopin C., Cornet J., Smith-Ravin J., Rochefort K., Leroi F. (2018). Effect of Vacuum and Modified Atmosphere Packaging on the Microbiological, Chemical and Sensory Properties of Tropical Red Drum (*Sciaenops ocellatus*) Fillets Stored at 4 °C. Int. J. Food Microbiol..

